# Temporenzia

**Published:** 1888-01

**Authors:** D. L. Cetlinski

**Affiliations:** 232 West 46th Street, New York


### TEMPORENZIA.

If there is anything that puzzles my mind it is the temperance movement. In the first place I find the very name by which it goes to be a philological problem.

Temperance means *moderation.* Este moclus in rebus. While the apostles of temperance preach total abstinence. Yet, as we live in a free country where law is used as food for law pettifoggers and dictionary products to clothe the poor thoughts of our popular elocutionists, that philological problem is easily solved. A more difficult problem is presented to my mind by the principle which seems to be the vital force which moves the actors in the movement.

To remedy the pathological condition, cold intemperance by total abstinence, is evidently based on the old worn out Gallian theory of *contraria contraribus* or cure by opposites, in total ignorance of the true scientific principle of therapeutics of similia similibus, or cure by likes newly revealed by a German physician named Hahnemann, half a century ago and which theory has spread over the civilized world, and has forced our venerable Aesculapians to modify their so-called heroic treatment to a more reasonable proportion and even to mask their poisonous drugs with saccharine coating and make pills into *parvular.* It seems to have been forgotten that opposites produce worse re-action, and hence the numerous relapses among our converts to total abstinence. Another bad consequence of treating intemperance by contraria is a kind of hygienic hypocrisy, which among our relapsed converts is an unpardonable sin against ethics. It is true that some exceptional cases of cure of intemperance by total abstinence are well attested but these cures are mostly to be met among those individuals who are endowed with a high degree of will power and self-control, once awakened to the consciousness of their own redeeming power by the ceremony of taking the pledge under psychological influences : but I think that such individuals may be easier and more agreeably cured by our female inventors of what they call Christian Science, a name as charming as are some of the lovely practitioners of that so-called science.

Finally it seems to me advisable to remind our apostles of teetotalism that a very successful attempt was made some years ago in Sweden, to treat intemperance according to the principle of similia similibus as understood by those Swedish physicians. They impregnate all articles of food given to the inebriate patients with the very stuff they are hankering after. Their intention is laudable as it may create a disgust in the inebriates for stimulants but this method is not what is called homeopathy: it is known by the name of *isopathy* or cure by sameness, and it reminds us of the famous lines,

"'Tis true a scorpion oil is said
To cure the wounds the vermin make."

Let us hope, nevertheless. that time will reveal the true homeopathic remedy for the mind and body, undermining poison of intemperance in all things.

D. L. Cetlinski, M. D.,

232 West 46th Street, New York.

